# Involving male partners in maternity care in Burkina Faso: a randomized controlled trial

**DOI:** 10.2471/BLT.17.206466

**Published:** 2018-06-04

**Authors:** Marina AS Daniele, Rasmané Ganaba, Sophie Sarrassat, Simon Cousens, Clémentine Rossier, Seydou Drabo, Djeneba Ouedraogo, Veronique Filippi

**Affiliations:** a The London School of Hygiene & Tropical Medicine, Keppel Street, Bloomsbury, London, WC1E 7HT, England.; b AfricSanté, Bobo-Dioulasso, Burkina Faso.; c Institut de démographie et socioéconomie, University of Geneva, Geneva, Switzerland.; d Faculty of Medicine, University of Oslo, Oslo, Norway.

## Abstract

**Objective:**

To determine whether an intervention to involve the male partners of pregnant women in maternity care influenced care-seeking, healthy breastfeeding and contraceptive practices after childbirth in urban Burkina Faso.

**Methods:**

In a non-blinded, multicentre, parallel-group, superiority trial, 1144 women were assigned by simple randomization to two study arms: 583 entered the intervention arm and 561 entered the control arm. All women were cohabiting with a male partner and had a low-risk pregnancy. Recruitment took place at 20 to 36 weeks’ gestation at five primary health centres in Bobo-Dioulasso. The intervention comprised three educational sessions: (i) an interactive group session during pregnancy with male partners only, to discuss their role; (ii) a counselling session during pregnancy for individual couples; and (iii) a postnatal couple counselling session. The control group received routine care only. We followed up participants at 3 and 8 months postpartum.

**Findings:**

The follow-up rate was over 96% at both times. In the intervention arm, 74% (432/583) of couples or men attended at least two study sessions. Attendance at two or more outpatient postnatal care consultations was more frequent in the intervention than the control group (risk difference, RD: 11.7%; 95% confidence interval, CI: 6.0 to 17.5), as was exclusive breastfeeding 3 months postpartum (RD: 11.4%; 95% CI: 5.8 to 17.2) and effective modern contraception use 8 months postpartum (RD: 6.4%; 95% CI: 0.5 to 12.3).

**Conclusion:**

Involving men as supportive partners in maternity care was associated with better adherence to recommended healthy practices after childbirth.

## Résumé

**Objectif:**

Déterminer si une intervention visant à faire participer le compagnon des femmes enceintes aux soins de maternité a permis d'influencer le recours aux soins, l'allaitement et les pratiques contraceptives après l'accouchement dans une ville du Burkina Faso.

**Méthodes:**

Dans le cadre d'un essai de supériorité en groupes parallèles, multicentrique, non aveugle, 1144 femmes ont été réparties par randomisation simple en deux groupes d'étude: 583 dans le groupe expérimental et 561 dans le groupe témoin. Toutes les femmes vivaient avec un compagnon et avaient une grossesse à faible risque. Leur recrutement a eu lieu entre 20 et 36 semaines de grossesse dans cinq centres de soins primaires de Bobo-Dioulasso. L'intervention comprenait trois séances pédagogiques: (i) une séance de groupe interactive pendant la grossesse, avec les compagnons uniquement, afin de discuter de leur rôle; (ii) une séance de conseil pendant la grossesse pour les couples; et (iii) une séance de conseil postnatale pour les couples. Le groupe témoin a bénéficié uniquement de la prise en charge habituelle. Nous avons suivi les participants à 3 et 8 mois après l'accouchement.

**Résultats:**

Le taux de suivi était supérieur à 96% aux deux visites de suivi. Dans le groupe expérimental, 74% (432/583) des couples ou des hommes avaient assisté à au moins deux des séances prévues. La présence à deux consultations postnatales externes ou plus était plus fréquente dans le groupe expérimental que dans le groupe témoin (différence de risques, DR: 11,7%; intervalle de confiance de 95%, IC: 6,0 à 17,5), comme l'allaitement exclusif 3 mois après l'accouchement (DR: 11,4%; IC 95%: 5,8 à 17,2) et l'utilisation de moyens de contraception modernes efficaces 8 mois après l'accouchement (DR: 6,4%; IC 95%: 0,5 à 12,3).

**Conclusion:**

La participation et l'accompagnement des hommes aux soins de maternité étaient associés à un meilleur suivi des pratiques recommandées après l'accouchement.****

## Resumen

**Objetivo:**

Determinar si una intervención para involucrar a las parejas masculinas de las mujeres embarazadas en la atención de la maternidad influyó en la búsqueda de atención, la lactancia materna saludable y las prácticas anticonceptivas después del parto en una zona urbana de Burkina Faso.

**Métodos:**

En un ensayo preponderado, no cegado, multicéntrico, de grupos paralelos, se asignó a 1144 mujeres aleatorias a dos grupos del estudio: 583 entraron en el grupo de intervención y 561 entraron en el grupo de control. Todas las mujeres convivían con una pareja masculina y tenían un embarazo de bajo riesgo. La selección tuvo lugar entre las semanas 20 y 36 de gestación en cinco centros de salud primarios en Bobo-Dioulasso. La intervención contó con tres sesiones educativas: (i) una sesión grupal interactiva durante el embarazo sólo con las parejas masculinas, para hablar sobre su papel; (ii) una sesión de asesoramiento durante el embarazo para parejas individuales; y (iii) una sesión de consejería posnatal en pareja. El grupo de control recibió sólo atención rutinaria. Seguimos a los participantes a los 3 y 8 meses después del parto.

**Resultados:**

La tasa de seguimiento fue superior al 96% en dos visitas. En el grupo de intervención, el 74% (432/583) de parejas u hombres asistieron al menos a dos sesiones de estudio. La asistencia a dos o más consultas de atención posnatal para pacientes ambulatorios fue más frecuente en el grupo de intervención que el grupo de control (diferencia de riesgo, RD: De 1,7% a 95% en el intervalo de confianza, IC: De 6,0 a 17,5), en la lactancia materna exclusiva 3 meses después del parto (RD: De 11,4% a 95% IC: De 5,8 a 17,2) en anticonceptivos modernos efectivos 8 meses después del parto (RD: De 6,4% a 95% IC: De 0,5 a 12,3).

**Conclusión:**

Involucrar a los hombres como apoyo en la atención de maternidad se asoció con una mejor adherencia a las prácticas recomendadas de salud después del parto.

## ملخص

**الهدف:**

تحديد ما إذا كان التدخل لإشراك الشركاء الذكور للنساء الحوامل في رعاية الأمومة، قد أثر على طلب الرعاية الصحية والرضاعة الطبيعية وممارسات منع الحمل بعد الولادة في المناطق الحضرية في بوركينا فاصو.

**الطريقة:**

في تجربة متفوقة غير مغشاة، متعددة المراكز، في مجموعات متوازية، تم اختيار 1144 امرأة بطريقة عشوائية بسيطة للمشاركة في فرعي الدراسة: 583 منهن انضممن لفرع التدخل، بينما انضم 561 منهن لفرع التحكم. كانت كل السيدات تعيشن مع شريك من الذكور، وكانت احتمالات الحمل منخفضة الخطورة. وتمت الاختيارات في الفترة من 20 إلى 36 أسبوعاً من الحمل في خمسة مراكز صحية أولية في بوبو ديولاسو. وتألف التدخل من ثلاث جلسات تعليمية: (أ) جلسة جماعية تفاعلية أثناء الحمل مع الشركاء الذكور فقط، لمناقشة دورهم؛ و(ب) جلسة المشورة خلال فترة الحمل لكل زوجين على حدة؛ و(ج) جلسة المشورة للزوجين بعد الولادة. لم تحصل مجموعة التحكم إلا على الرعاية الروتينية المعتادة فقط. وقمنا بمتابعة المشاركين بعد مرور 3 و8 أشهر من الولادة.

**النتائج:**

كان معدل المتابعة أكثر من 96٪ في كلتا المرتين. بالنسبة لفرع التدخل ، حضر 74٪ (432/583) من الزوجين أو الرجال جلستين دراسيتين على الأقل. وكان الحضور في اثنتين أو أكثر من استشارات رعاية ما بعد الولادة في العيادات الخارجية، أمراً متكرر الحدوث بشكل أكبر في مجموعة التدخل أكثر منه في مجموعة التحكم (فرق المخاطر: 11.7٪؛ فاصل الثقة 95٪، 6.0 إلى 17.5)، كما كانت الرضاعة الطبيعية الحصرية لمدة 3 أشهر بعد الولادة (فرق المخاطر: 11.4٪؛ فاصل الثقة 95٪: 5.8 إلى 17.2)، واستخدام وسائل منع الحمل الحديثة الفعالة لمدة 8 أشهر بعد الولادة (فرق المخاطر 6.4٪؛ فاصل الثقة 95٪: 0.5 إلى 12.3).

**الاستنتاج:**

ارتبط إشراك الرجال باعتبارهم شركاء داعمين في رعاية الأمومة بمستوى أفضل من الالتزام بالممارسات الصحية الموصى بها بعد الولادة.

## 摘要

**目的:**

在布基纳法索 (Burkina Faso) 城市地区，确定孕产期保健中孕产妇男性伴侣的干预是否会影响分娩后的就医行为、健康的母乳喂养和避孕行为。

**方法:**

在一个采用非盲法、多中心、平行组的优势试验中，将 1144 名女性简单随机分为两个研究组：583 名分入干预组，561 名分入控制组。所有女性都与男性伴侣同居并且妊娠风险低。在博博迪乌拉索 (Bobo-Dioulasso) 五个主要的健康中心进行招募，共招募了妊娠期在 20 至 36 周的被试者。干预包含三个不同层次的教育型会谈：(i) 在妊娠期间仅与男性伴侣进行互动式小组会谈以讨论其职责；(ii) 在妊娠期间对每一对夫妇进行咨询会谈；以及 (iii) 产后夫妇咨询会谈。控制组仅接受常规护理。我们分别在产后第 3 个月和第 8 个月对参与者的情况进行了随访。

**结果:**

两次的随访率均超过 96%。在干预组中，74% (432/583) 的夫妇或男性参与了至少两次的学习会谈。干预组参与两次或以上的门诊产后保健咨询的频率高于控制组（风险差异：11.7%；95% 置信区间，CI：6.0 至 17.5)，如在产后 3 个月进行纯母乳喂养（风险差异：11.4；95% 置信区间，CI：5.8 至 17.2）以及产后 8 个月有效的现代避孕方法（风险差异：6.4%；95% 置信区间，CI：0.5 至 12.3)。

**结论:**

让男性作为支持伴侣参与到孕产期保健中关系到孕妇分娩后更好地遵循推荐的健康习惯。

## Резюме

**Цель:**

Определить, повлияло ли вмешательство путем вовлечения мужчин, являющихся партнерами беременных женщин, в систему охраны материнства на активное обращение за медицинской помощью, на приверженность здоровым практикам грудного вскармливания и на использование контрацепции после родов в городских районах Буркина-Фасо.

**Методы:**

В открытом многоцентровом параллельном групповом исследовании эффективности 1144 женщины были разделены путем простой рандомизации на две группы: 583 вошли в группу вмешательства, 561 — в контрольную группу. Все женщины сожительствовали с мужчиной-партнером и имели низкий риск осложнений при беременности. Участниц набирали на сроке беременности от 20 до 36 недель в пяти центрах первичной медико-санитарной помощи в Бобо-Диуласо. Вмешательство состояло из трех образовательных занятий: (i) интерактивное групповое занятие во время беременности только с партнерами-мужчинами для обсуждения их роли; (ii) консультирование пар во время беременности; (iii) консультирование пар после родов. Контрольная группа получала стандартную медицинскую помощь. Участники проходили последующее наблюдение через 3 и 8 месяцев после родов.

**Результаты:**

Доля пациентов, оставшихся под наблюдением, составляла более 96% в обоих случаях. В группе вмешательства 74% (432/583) пар или мужчин посетили не менее двух занятий в рамках исследования. Посещение двух или более амбулаторных консультаций по послеродовому уходу было более частым в группе вмешательства, чем в контрольной группе (разность рисков, РР: 11,7; 95%-й ДИ: от 6,0 до 17,5), а также исключительно грудное вскармливание через 3 месяца после родов (РР: 11,4; 95%-й ДИ: от 5,8 до 17,2) и эффективное использование современных контрацептивов через 8 месяцев после родов (РР: 6,4; 95%-й ДИ: от 0,5 до 12,3).

**Вывод:**

Вовлечение мужчин в качестве партнерской поддержки в систему охраны материнства было связано с лучшей приверженностью рекомендованным здоровым практикам после родов.

## Introduction

Ending preventable maternal and perinatal mortality necessarily involves engaging with families and communities.^1^ Male partners, in particular, exert a considerable influence on women’s use of reproductive health services and participate in decisions that affect health outcomes.^2^ Surveys from sub-Saharan Africa show that most women with a male partner would be willing for him to participate in maternity care, except where there is a concern about domestic violence, alcohol abuse or disclosing human immunodeficiency virus (HIV) infection status.^3^^,^^4^ However, few men join their pregnant partners during antenatal or postnatal appointments at health-care facilities, often because of the perception that this is not their role.^5^^,^^6^ Moreover, the clinic’s infrastructure may not be suitable for couples, there may be concerns about congestion or privacy and opening hours may be inconvenient.^7^^,^^8^ Staff attitudes can also be a problem.^9^ Where policies to invite male partners to antenatal care appointments have been introduced, the focus has tended to be on HIV testing, after which men may be told to leave.^10^

In the last few decades, strategies promoting male involvement in reproductive health services have received increasing attention, such as endorsement by the World Health Organization.^11^ Although systematic reviews conclude that these strategies can improve care-seeking throughout the childbearing period, most evidence comes from observational studies or evaluations of complex interventions that were not specifically designed to investigate male involvement.^12^^–^^16^ Consequently, the impact of these strategies is not clear. Few high-quality experimental studies have been conducted in sub-Saharan Africa and even fewer have assessed facility-based interventions,^17^^,^^18^ apart from those focusing on the prevention of mother-to-child HIV transmission.^19^

Burkina Faso has high maternal and infant mortality.^20^ Although the majority of women give birth in health-care facilities (the latest estimate was 66% in 2010), most do not have regular check-ups postpartum.^20^^,^^21^ Even in urban areas, fewer than half attend the recommended two outpatient postnatal consultations.^22^^,^^23^ Moreover, fewer than half of infants are exclusively breastfed 3 months postpartum.^20^ One quarter of women of reproductive age have an unmet need for family planning and few initiate contraception promptly following childbirth.^23^^,^^24^ These health vulnerabilities reflect women’s social and economic disadvantages in a country that is characterized by patriarchal family structures, polygyny and women marrying older men.^20^ Although childbearing and the care of young children are considered female domains, men are usually the ultimate decision-makers on care-seeking.^25^^,^^26^ However, male partners are rarely seen in health-care facilities and have scarcely any contact with health workers, which limits their exposure to health information.^22^^,^^23^ Older women, especially the male partner’s mother, are regarded as experts on infant care and feeding.^27^ Traditionally, in addition to breast milk, neonates in Burkina Faso receive water and herbal infusions.^27^ Opposition to contraception by the male partner is often cited as an obstacle and is associated with lower contraception use by women.^28^^,^^29^ Two community-based projects involving men have been initiated in the country but rigorous evaluations have not been published.^30^^,^^31^

The aim of our study was to determine whether an intervention designed to involve the male partners of pregnant women in Burkina Faso in facility-based maternity care influences care-seeking and healthy practices after childbirth. Our hypothesis was that the intervention would increase postnatal care attendance, the duration of exclusive breastfeeding and the use of postpartum contraception.

## Methods

We performed an individually randomized, multicentre, superiority trial. Participants were enrolled at the five largest primary health centres in one of three health districts in the city of Bobo-Dioulasso – each health centre served a predominantly urban population of around 20 000 and offered antenatal, labour and birth, postnatal and family planning services.^32^ In 2014, an average of 66 births took place every month in each study health centre.^32^ Maternity staff were mostly female: the majority were auxiliary midwives (i.e. *accoucheuses auxiliaires*) and the minority were midwives (i.e. *sage femmes* or *maïeuticiens d’état*). Women who had obstetric complications or required a caesarean section were referred to the local district or university hospital, a maximum of 4 km distance.

In this setting, almost all women attended antenatal care at least once.^20^ We invited pregnant women who were attending routine check-ups to participate in the study. Eligible women were aged between 15 and 45 years, cohabiting with a man (regardless of marital status), pregnant with an estimated gestational age of 20 to 36 weeks and, based on their obstetric risk profile, expected to be able to give birth in a primary health centre. We excluded women who were recommended at the time of recruitment to give birth in a referral hospital. We assigned participants to the intervention or control arm of the study on a 1:1 basis by simple, nonstratified randomization according to a sequence generated by the principal investigator using the random integer function of a scientific calculator. The principal investigator prepared sealed opaque envelopes containing participants’ allocation and study identification number. At randomization, research assistants invited participants to select an envelope.

Women allocated to the intervention group and their male partners were invited to participate in three 1-hour educational sessions in French or a local language at their primary health centre, delivered by auxiliary midwives and midwives who attended a 1-day training workshop. The sessions comprised: (i) an interactive group session between 20 weeks’ gestation and term for male partners only, to discuss men’s role; (ii) a counselling session between 20 weeks’ gestation and term for each couple individually to provide information and advice on topics related to pregnancy, birth, the postpartum period and family planning; and (iii) a postnatal couple counselling session before postpartum discharge, to discuss further the postpartum period and family planning. Participants were invited by several means, including letters and follow-up phone calls. The intervention is described in detail in Box 1. Women in both study arms received routine maternity care, in which male partners normally participate very rarely.

Box 1 **The study intervention to involve male partners in maternity care, Burkina Faso, 2015–2016**

The intervention consisted of three components: (i) an interactive group discussion session for male partners only; (ii) an individual couple counselling session during pregnancy; and (iii) a postnatal couple counselling session before discharge from the facility.

All sessions took place in a participating primary health-care centre. The health workers who delivered the intervention were auxiliary midwives or midwives. These workers had all attended formal training courses to Burkina Faso Ministry of Health standards and generally provided complete care for low-risk pregnant women and neonates. For this study, they attended a 1-day training workshop on working with men and couples, which included discussions, role-playing and troubleshooting on gender issues, particularly on women’s control over their male partner’s involvement. Dedicated in-work support and quality control were in place for the duration of the study. On average, 23 health workers participated at each facility.

Each session lasted approximately 1 hour. Each couple or man was invited to attend each individual session once. The first two sessions took place as soon as possible after the woman was recruited into the study (i.e. any time between 20 weeks’ gestation and term). At recruitment, participants in the intervention group received an invitation letter for the first session, which they passed on to their male partners. The invitation was reiterated in a phone call from a health worker a few days later.

**Interactive group discussions**

Group discussions took place every Saturday morning in an open-air meeting space at each primary health-care centre. Between two and five health workers conducted the sessions in French and local languages. A total of 52 sessions were conducted, each attended by 3 to 13 men. Health workers checked the men’s names on arrival against a list of those who had been invited.

During the sessions, health workers stimulated discussions by reading out the stories of three fictional couples who were having a baby. In these stories, adverse events occurred when there was no communication or collaboration between the man and woman or when they lacked adequate health information. With both good communication and information, there was a positive outcome. Participants were encouraged to reflect critically on their roles as men and partners. A guide for conducting the group sessions was drawn up by the principal investigator. The content was entirely original. At the end of these sessions, men were invited to attend the first couple counselling session and were given 1000 CFA francs (equivalent to 1.70 United States dollars at the time) as a one-off contribution to travel expenses.

**Couple counselling sessions during pregnancy**

The purpose of the couple counselling sessions was to provide information and advice to both partners on a range of topics related to pregnancy, birth and the postpartum period, including: (i) the importance of antenatal and postnatal care; (ii) birth preparedness and signs of labour; (iii) danger signs for the mother and newborn child; (iv) exclusive breastfeeding; (v) the healthy timing and spacing of pregnancies; and (vi) postpartum contraception.

Sessions took place in a private consultation room with one or two health workers. They were interactive and questions were encouraged. Health workers used a flipchart, which contained illustrations on the side facing the participants and related text on the side facing the health worker. The chart was adapted from two existing counselling tools produced by the World Health Organization and the Ministry of Health of Senegal.^33^^,^^34^

When the conversation moved to family planning, the focus was on each couple’s situation and reproductive intentions. Samples of contraceptive devices were available to see and touch. Couples were given the opportunity to consult each other and express their choice of contraceptive method for use after the birth. If appropriate, a simple, non-binding plan for the initiation of contraception was drawn up and documented in the woman’s health booklet.

**Postnatal couple counselling sessions**

If a woman in the intervention arm gave birth in a primary health-care centre, the couple was invited to another counselling session. This usually took place around 6 hours after giving birth, following the predischarge physical examination. In routine care, women are given health advice at this time without their partners, either alone or in groups. Attempts were made to reach the male partner by phone if he was not in the facility. This session was a further opportunity to discuss and provide information relevant to the weeks and months after birth. If the couple had not yet decided about contraception, they had the opportunity to do so during this session, with the option of immediately initiating some methods or getting a prescription before discharge. The same flipchart was used as in the first couple counselling session.

The primary study outcomes were: (i) the woman’s attendance at two or more scheduled, outpatient, postnatal care consultations in the 6 weeks after birth; (ii) exclusive breastfeeding 3 months postpartum; and (iii) the use of effective modern contraception (i.e. implants, intrauterine devices, injectable and oral contraceptives, and permanent methods) 8 months postpartum. Secondary outcomes were: (i) use of a long-acting or permanent method of contraception (i.e. intrauterine devices, implants and female or male sterilization) 8 months postpartum; (ii) use of any contraceptive or contraceptive method, including less effective methods, 3 and 8 months postpartum; (iii) the timely initiation of effective, modern contraception within a period during which conception was reasonably unlikely; (iv) unmet need for contraception 8 months postpartum; (v) good relationship adjustment 8 months postpartum; and (vi) complete satisfaction with routine care. We determined good relationship adjustment from the woman’s satisfaction with the relationship and the degree of communication, shared decision-making and agreement between the couple on key reproductive health issues. This outcome was assessed using an unvalidated tool adapted from existing instruments.^35^^,^^36^ We assessed satisfaction with routine care using an unvalidated tool developed from existing instruments.^37^^,^^38^ Details are given in Box 2 and associated Table 1.

Box 2 **Study outcomes in the intervention to involve male partners in maternity care, Burkina Faso, 2015–2016**

**Primary outcomes**

(i) The woman’s attendance at two or more scheduled, outpatient, postnatal care consultations

A woman was classed as having attended scheduled, postnatal care if she had attended at least two consultations in the first 6 weeks after giving birth – the minimum recommended by the national protocol.^39^ These usually took place 6 days and 6 weeks postpartum.

(ii) Exclusive breastfeeding 3 months postpartum

Because the duration of exclusive breastfeeding in Burkina Faso is usually short, we decided that an increase in the proportion of women who were exclusively breastfeeding 3 months postpartum would constitute a meaningful public health gain. The definition of exclusive breastfeeding was based on WHO criteria.^40^ The mother was read a list of food and drink items and breastfeeding was classed as exclusive if the infant had received food or drink other than breast milk only once or twice.

(iii) Use of effective modern contraception 8 months postpartum

We defined an effective modern contraceptive method as one that had an unintended pregnancy rate of 10% or less per year, as commonly employed.^41^ The methods available locally were implants, intrauterine devices, injectable and oral contraceptives, and permanent methods.

**Secondary outcomes**

(i) Use of long-acting or permanent methods of contraception 8 months postpartum

This was defined as the proportion of women who were using intrauterine devices or contraceptive implants, who had undergone sterilization or whose partner had undergone sterilization by 8 months postpartum.

(ii) Use of any contraceptive or contraceptive method 3 and 8 months postpartum

The methods read out by the interviewer were: (i) male and female sterilization; (ii) intrauterine devices; (iii) injectable contraceptives; (iv) contraceptive implants; (v) oral contraceptives; (vi) male and female condoms; (vii) the rhythm method; (viii) the lactational amenorrhea method; (ix) withdrawal; and (x) the standard days method. Other reported methods, including traditional methods, were also included.

(iii) Timely initiation of effective modern contraception

The initiation of an effective modern contraceptive method, which were those listed in primary outcome (iii), was defined as timely if it took place within a period during which conception was reasonably unlikely. Table 1 (available at: http://www.who.int/bulletin/volumes/95/7/17-206466) below lists specific criteria, which are based on the time during which lactational amenorrhoea provides 98% protection against unwanted pregnancy.^42^

(iv) Unmet need for contraception 8 months postpartum

We used the revised definition of unmet need provided by the Demographic and Health Survey organization.^43^

(v) Good relationship adjustment 8 months postpartum

Relationship adjustment was determined from the woman’s satisfaction with her relationship with her partner and the degree of communication, shared decision-making and agreement between the couple on key issues related to reproductive health. These factors are plausible mechanisms through which interventions to involve men may act to improve care-seeking and other behavioural outcomes.^12^ Our unvalidated tool for assessing this outcome was adapted from existing instruments, including the Dyadic Adjustment Scale and the Locke–Wallace Marital Adjustment Test.^35^^,^^36^

(vi) Complete satisfaction with routine care

To determine whether being in the intervention group adversely or positively affected the woman’s experience of routine care, we used an unvalidated measurement tool for satisfaction, which was developed by adapting questions from existing instruments.^37^^,^^38^ To ensure comparability between the two study arms, the questions asked did not refer to the care received as part of the intervention.

**Table 1:** Criteria for assessing timely initiation of effective modern contraception

Situation at time of contraception initiation	Timely initiation of effective modern contraception	
	Contraception initiated ≤ 6 months postpartum and exclusive breastfeeding at 3 months	Contraception initiated > 6 months postpartum or not exclusively breastfeeding at 3 months
Amenorrhoea and abstinence	Yes	Yes
Amenorrhoea and sexually active	Yes	No
Menses returned and abstinence	Yes	Yes
Menses returned and sexually active	No	No

We collected baseline data through interviews at enrolment using a questionnaire on the women’s demographic and socioeconomic characteristics, including age, parity, ethnicity, religion, occupation and educational level, on their reproductive health history and on their male partner’s characteristics. During follow-up interviews with the women at home, we collected data on health and behavioural outcomes 3 and 8 months postpartum. Their male partners were not interviewed. All questionnaires were in French, which was translated into local languages (i.e. Dioula and Moore) verbally. We conducted field trials of the questionnaires with nonparticipating women attending the study centres. To assess compliance with the study arm assignment and adherence to the intervention, participants’ names and identification numbers were recorded at each study session.

### Statistical analysis

We tabulated baseline data using descriptive statistics and any major differences between study arms were identified by visual inspection. For primary and secondary outcomes, we tested the null hypothesis that the intervention had no effect in intention-to-treat analyses. These outcomes were treated as binary variables and we assessed the intervention’s effect using generalized linear models with the Bernoulli/binomial family of distributions and the identity link. We report the magnitude of the effect as the risk difference (RD) between intervention and control arms, with 95% confidence intervals (CIs). To account for the possible effect of the study site, we included the recruitment primary health centre as a fixed effect in the generalized linear model and all effect estimates reported were adjusted for this variable. In addition, the possibility that the effect of the intervention varied across sites was also explored by performing likelihood ratio tests on the study’s results stratified by primary health-care centre.

We calculated that that a sample size of 1115 would be sufficient to detect an increase in the percentage of women attending the recommended number of postnatal consultations from 30 to 39%. Such sample size would also be sufficient to detect an increase from 25 to 34% in the proportion of women who were still exclusively breastfeeding 3 months postpartum and an increase from 20 to 28% in the proportion of women using effective modern contraception 8 months postpartum. All calculations assumed 95% CIs and 80% power and allowed for a 20% loss to follow-up.

We could not blind health workers and session attendees to the intervention. However, interviewers collected baseline data before carrying out randomization and can thus be considered blinded during that phase, as were all data entry staff. Outcome data collectors were probably blinded to the study allocation but that could not be guaranteed. It was not feasible for the principal investigator to be blinded during the analysis.

Participants gave written informed consent of their own free will. The study was fully compliant with the ethical principles of the World Medical Assembly Declaration of Helsinki as amended by the 59^th^ General Assembly in 2008. Ethical approval was obtained from the research ethics committee of the London School of Hygiene & Tropical Medicine, the institutional review board of the Population Council and the health research ethics committee of the Ministry of Health in Burkina Faso. The trial was registered on ClinicalTrials.gov (NCT02309489).

## Results

**Fig. 1:**
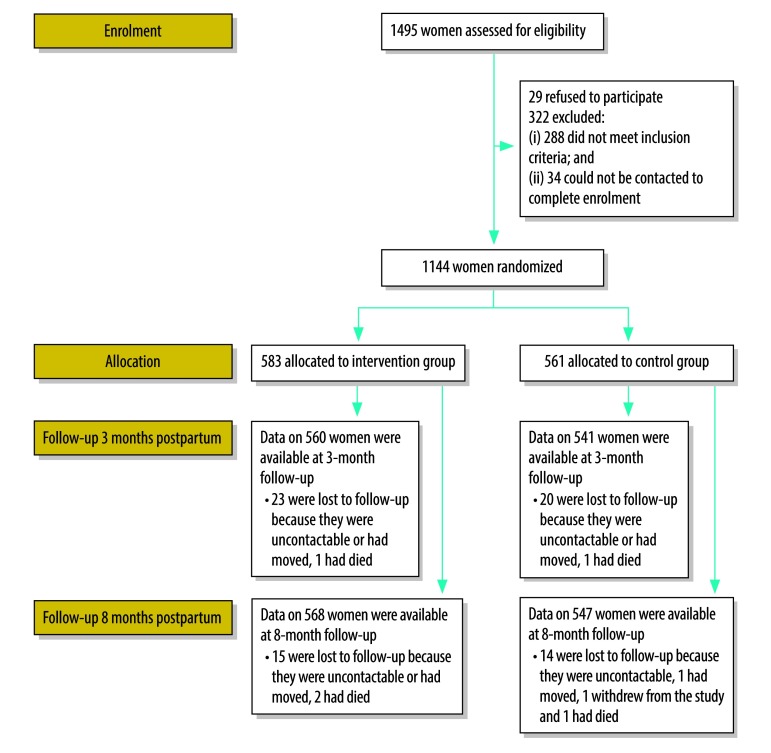
Flow diagram, intervention to involve male partners in maternity care, Burkina Faso, 2015–2016

We recruited 1144 women between 16 February and 12 June 2015: 583 were randomized to the intervention arm and 561 to the control arm (Fig. 1). The follow-up rate was over 96% both 3 and 8 months postpartum. Follow-up ended on 4 July 2016. There was no substantial difference in baseline characteristics between the study arms: the women’s and their partners’ sociodemographic characteristics are shown in Table 2 and Table 3, respectively, and the women’s obstetric and contraception history is shown in Table 4.

**Table 2:** Women’s sociodemographic characteristics, intervention to involve male partners in maternity care, Burkina Faso, 2015–2016

Characteristic	No. of women (%)^a^	
	Intervention group (*n* = 583)	Control group (*n* = 561)
**Recruitment health centre**		
Bolomakote	89 (15.3)	86 (15.3)
Guimbi	101 (17.3)	109 (19.4)
Ouezzinville	163 (28.0)	165 (29.4)
Sarfalao^b^	119 (20.4)	92 (16.4)
Secteur 24	111 (19.0)	109 (19.4)
**Age in years, mean (SD)**	26.3 (6.0)	26.3 (5.9)
**Age, years**		
15–19	73 (12.5)	75(13.4)
20–24	179 (30.7)	164 (29.2)
25–29	163 (28.0)	158 (28.2)
30–34	109 (18.7)	99 (17.7)
35–39	46 (7.9)	56 (10.0)
40–45	13 (2.2)	9 (1.6)
**Ethnic group**		
Bobo or Bwa	108(18.5)	110 (19.6)
Dagara, Lobi, Birifor, Djan and similar	61 (10.5)	45 (8.0)
Dioula, Dafing, Samo and similar	93 (16.0)	85 (15.2)
Gourounsi, Ko or Nounouma	24 (4.1)	24 (4.3)
Mossi, Gourmanche, Bissa and similar	260 (44.6)	263 (46.9)
Peulh	16 (2.7)	19 (3.4)
Other	21 (3.6)	15 (2.7)
**Religion^c^**		
Muslim	420 (72.2)	407 (72.6)
Christian	158 (27.2)	144 (25.7)
Traditional or animist	1 (0.2)	5 (0.9)
No religion	3 (0.5)	5 (0.9)
**Educational level**^c^		
No education	311 (53.3)	278 (49.6)
Primary school	145 (24.9)	168 (30.0)
Above primary school	126 (21.6)	115 (20.5)
**Type of occupation^c,d^**		
No work outside the home	232 (39.8)	213 (38.0)
Street vendor	246 (42.3)	254 (44.0)
Craftswoman	52 (8.9)	35 (6.2)
Shopkeeper	39 (6.7)	41 (7.3)
Other	22 (4.0)	26 (4.6)

SD: standard deviation.

^a^ All values in the table represent absolute numbers and percentages unless otherwise stated.

^b^ The difference between the number of participants assigned to the intervention and control groups in the Sarfalao health centre was due to an isolated incident in which a data collector initially used a batch of randomization envelopes that had not been mixed and that assigned all participants to the intervention. Once this was noticed, the batch was immediately replaced. This error did not bias the allocation.

^c^ Data were missing for one woman in the intervention group.

^d^ Percentages for occupations add up to more than 100% as more than one occupation was allowed.

**Table 3:** Male partners’ sociodemographic characteristics, intervention to involve male partners in maternity care, Burkina Faso, 2015–2016

Characteristic^a^	No. of men (%)^b,c^	
	Intervention group (*n* = 583)	Control group (*n* = 561)
**Age in years, mean (SD)**	40.1 (18.8)	40.6 (20.3)
**Age, years^d^**		
20–29	126 (23.6)	138 (27.4)
30–39	275 (51.6)	246 (48.8)
40–49	105 (19.7)	101 (20.0)
≥ 50	27 (5.1)	19 (3.8)
**Age difference between man and woman in years, median**	8	7
**Educational level^e^**		
No education	247 (48.4)	244 (48.3)
Primary school	134 (26.3)	125 (24.8)
Above primary school	129 (25.3)	136 (26.9)
**Type of occupation^f^**		
Agriculture	44 (7.6)	58 (10.3)
Street vending	124 (21.3)	110 (19.6)
Skilled manual labour	238 (40.8)	217 (38.7)
Shopkeeper or commerce	100 (17.2)	115 (20.5)
Public sector	41 (7.0)	41 (7.3)
Other	80 (13.7)	68 (12.1)
**Type of marriage^g^**		
Monogamous	504 (86.6)	476 (84.9)
Polygamous	78 (13.4)	85 (15.2)
**Person responsible for decisions on household expenses^g^**		
Woman	1 (0.2)	0 (0.0)
Male partner	491 (84.2)	474 (84.5)
Couple together	32 (5.5)	36 (6.4)
Third person	49 (8.4)	44 (7.8)
It depends or not sure	10 (1.7)	6 (1.0)
**Person responsible for the decision to seek health care^h^**		
Woman	2 (0.3)	3 (0.5)
Male partner	523 (89.7)	500 (89.1)
Couple together	38 (6.5)	39 (7.0)
Third person	19 (3.3)	13 (2.3)
It depends or not sure	1 (0.2)	5 (0.9)

SD: standard deviation.

^a^ The male partners’ characteristics were reported by the women.

^b^ All values in the table represent absolute numbers and percentages unless otherwise stated.

^c^ Percentages are of the total number of men for whom data were available in each category (e.g. age).

^d^ Data on age were missing for 50 men in the intervention arm and 57 in the control arm.

^e^ Data on educational level were missing for 73 men in the intervention arm and 56 in the control arm.

^f^ Percentages for occupations add up to more than 100% as more than one occupation was allowed.

^g^ Data on the person responsible for decisions on household expenses were missing for one man in the intervention arm.

^h^ Data on the person responsible for the decision to seek health care were missing for one man in the control arm.

**Table 4:** Women’s’ obstetric and contraception history, intervention to involve male partners in maternity care, Burkina Faso, 2015–2016

Obstetric and contraception history	No. of women (%)	
	Intervention group (*n* = 583)	Control group (*n* = 561)
**Parity**		
0	127 (21.8)	144 (25.7)
1	159 (27.3)	132 (23.5)
2	119 (20.4)	93 (16.6)
≥ 3	178 (30.5)	192 (34.2)
**Had ≥ 1 miscarriage or abortion**	91 (15.6)	107 (19.1)
**Had ≥ 1 stillbirth**	29 (5.0)	22 (3.9)
**Lost ≥ 1 child after birth**	96 (16.5)	106 (18.9)
**Nature of current pregnancy**		
Wanted	437 (75.0)	424 (75.6)
Mistimed	133 (22.8)	128 (22.8)
Not wanted	13 (2.2)	9 (1.6)
**Contraceptive methods used previously^a,b^**		
None	191 (32.8)	197 (35.1)
Male condom	69 (11.8)	64 (11.4)
Contraceptive pill	188 (32.3)	189 (33.7)
Injectable contraceptive	171 (29.3)	145 (25.8)
Implant	103 (17.7)	95 (16.9)
Other method	35 (6.0)	35 (6.2)
**Contraceptive users who did not inform their partner^b^**	58 (14.8)^c^	63 (17.3)^d^

^a^ The percentages for contraceptive methods used add up to more than 100% as more than one method could be mentioned.

^b^ Data were missing for one woman in the intervention arm and one in the control arm.

^c^ The denominator was 389: the number of women in the intervention group who ever used contraception.

^d^ The denominator was 360: the number of women in the control group who ever used contraception.

**Fig. 2:**
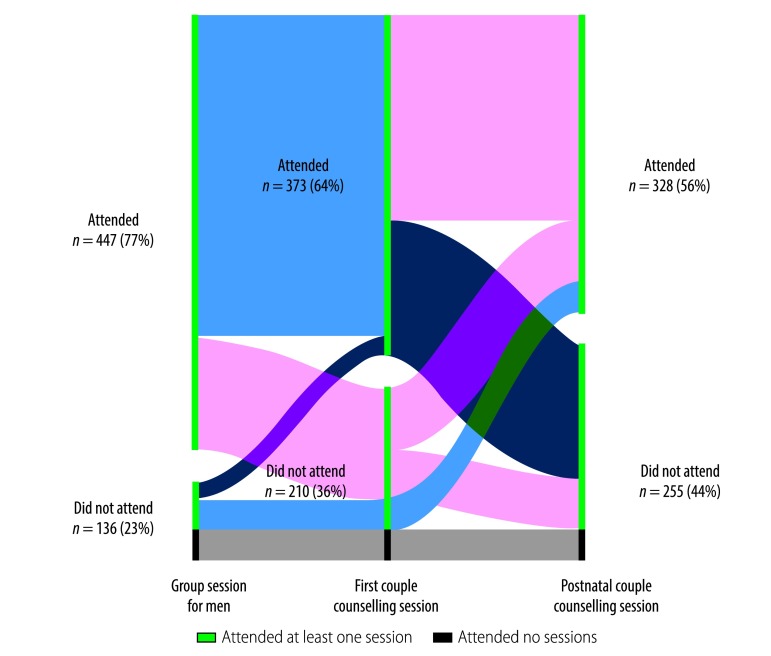
Attendance at educational sessions, intervention to involve male partners in maternity care, Burkina Faso, 2015–2016

Note: Percentages were calculated for the 583 women and their partners in the intervention group.

In the intervention arm, 37% (216/583) of couples or men attended all three educational sessions, 37% (216/583) attended two sessions, 17% (98/583) attended one and 9% (53/583) attended none. Thus, 74% (432/583) attended at least two sessions. No-one attended the same session more than once. As shown in Fig. 2, 77% (447/583) of male partners in the intervention group attended the group session for men, 64% (373/583) of couples attended the first couple counselling session and 56% (328/583) of couples attended the postnatal couple counselling session. In the intervention arm, 32% (187/583) of women gave birth in a referral hospital or another nonparticipating facility, very few of whom were referred, or transferred, from a primary health-care centre; the corresponding proportion in the control arm was 37% (208/561). This may explain why the postnatal couple counselling session was less well attended. There were two documented cases of noncompliance with arm assignment by men in the control group, which were due to communication errors and which resulted in them attending the group session for men.

As shown in Table 5, the intervention was associated with higher rates of attendance at two or more scheduled, outpatient, postnatal care consultations (RD: 11.7%; 95% CI: 6.0 to 17.5), of exclusive breastfeeding 3 months postpartum (RD: 11.4%; 95% CI: 5.8 to 17.2) and of effective modern contraception use 8 months postpartum (RD: 6.4%; 95% CI: 0.50 to 12.3). The intervention also had a positive effect on the use of long-acting contraception 8 months postpartum (RD: 8.1%; 95% CI: 2.9 to 13.4), on the use of any contraceptive method both 3 months (RD: 7.7%; 95% CI: 1.2 to 13.6) and 8 months (RD: 6.5%; 95% CI: 1.0 to 12.1) postpartum and on the timely initiation of effective modern contraception (RD: 7.6%; 95% CI: 0.2 to 15.1). The intervention was also associated with a reduction in unmet need for contraception 8 months postpartum (RD: −4.8%; 95% CI: −9.2 to −0.5). The increase in long-acting, reversible contraception use was almost entirely due to greater implant use (data not shown). No permanent methods were used. The intervention had a positive effect on the proportion of women with good relationship adjustment 8 months postpartum (RD: 8.7%; 95% CI: 2.9 to 14.6), but the proportion satisfied with routine care was not affected (RD: 0.4%; 95% CI: −4.8 to 5.6).

**Table 5:** Study outcomes, intervention to involve male partners in maternity care, Burkina Faso, 2015–2016

Outcome	Proportion of women, % (no./n)^a^		Intervention versus control group	
	Intervention group	Control group	Risk difference, % (95% CI)^b^	Risk ratio (95% CI)^b^
**Primary outcome**				
Woman’s attendance at ≥ 2 scheduled, outpatient, postnatal care consultations	61.1 (342/560)	49.0 (265/541)	11.7 (6.0 to 17.5)	1.23 (1.11 to 1.37)
Exclusive breastfeeding 3 months postpartum	43.4 (232/535)	31.5 (161/511)	11.4 (5.8 to 17.2)	1.35 (1.15 to 1.59)
Use of effective modern contraception 8 months postpartum	59.6 (330/554)	53.1 (283/533)	6.4 (0.5 to 12.3)	1.12 (1.01 to 1.24)
**Secondary outcome**				
Use of a long-acting or permanent method of contraception 8 months postpartum	30.7 (170/554)	22.9 (122/533)	8.1 (2.9 to 13.4)	1.33 (1.09 to 1.62)
Use of any contraceptive or contraceptive method 3 months postpartum	57.0 (315/553)	49.3 (262/532)	7.7 (1.2 to 13.6)	1.16 (1.04 to 1.30)
Use of any contraceptive or contraceptive method 8 months postpartum	70.6 (391/554)	64.4 (343/533)	6.5 (1.0 to 12.1)	1.10 (1.02 to 1.20)
Timely initiation of effective modern contraception	75.7 (249/329)	66.9 (188/281)	7.6 (0.2 to 15.1)	1.11 (1.00 to 1.24)
Unmet need for contraception 8 months postpartum	14.2 (79/560)	18.7 (101/539)	−4.8 (−9.2 to −0.5)	0.75 (0.57 to 0.98)
Good relationship adjustment 8 months postpartum	57.7 (323/560)	48.8 (263/539)	8.7 (2.9 to 14.6)	1.18 (1.05 to 1.32)
Complete satisfaction with routine care	73.8 (413/560)	73.0 (395/541)	0.4 (−4.8 to 5.6)	1.00 (0.94 to 1.08)

CI: confidence interval.

^a^ Number of participants who reported the outcome divided by the number for whom data on that specific outcome were available.

^b^ Adjusted by study recruitment primary health-care centre.

Tests for interaction indicated that the effect of the intervention varied across primary health-care centres for: (i) effective modern contraceptive use (*P* = 0.028); (ii) any contraceptive use 3 months (*P* = 0.026) and 8 months (*P* = 0.082) postpartum; and (iii) the timely initiation of effective modern contraception (*P* = 0.052). No individual facility appeared to perform consistently well or badly across all outcomes. At certain primary health centres we observed differences between the two study arms in some baseline characteristics, specifically the type of marriage, ethnicity, women’s education level and employment, parity and prior use of contraception. The results of the tests for interaction did not change when we included these characteristics in the models.

## Discussion

Our intervention to involve male partners in maternity care was associated with an increase in attendance at postnatal care consultations, in the duration of exclusive breastfeeding and in the use of postpartum contraception, especially long-acting, reversible contraception. The intervention also had a positive effect on communication between the couple and shared decision-making related to reproductive health. The proportion of participants who adopted the recommended behaviours increased between 6.4 and 11.7 percentage points for each of the three primary outcomes; for secondary outcomes, the improvement was between 4.8 and 8.7 percentage points. These results were achieved in the context of a high level of adherence to the intervention in an area where men are not traditionally involved in maternity care. Other trials in sub-Saharan Africa that involved inviting male partners into health-care facilities generally reported response levels below 50%.^17^^,^^44^^,^^45^ In our study, attendance was lowest for the postnatal counselling session, probably because one third of women chose to give birth in a nonparticipating referral hospital.

The intervention could have worked through several possible mechanisms. First, better communication between spouses and shared decision-making have been identified as enabling mechanisms in similar studies.^12^^,^^46^^,^^47^ In our study, couple counselling may have provided men and women with the opportunity to start conversations about issues they were not used to discussing openly. Moreover, in a context where men are seldom exposed to advice from health workers, the intervention may have enabled them to be better informed when participating in these conversations. Second, the male partner’s agreement may have encouraged women to choose long-acting, reversible contraception and removed known barriers, such as financial constraints and the fear that a disapproving husband might discover an implant’s insertion site.^23^ Third, men’s leverage with their own mothers may have helped some women to continue practicing exclusive breastfeeding and to refuse traditional supplementation with water and herbal infusions.^27^ Finally, more frequent postnatal contact with health workers probably reinforced the messages on exclusive breastfeeding and family planning.

This study has several limitations. Methodologically, our inability to guarantee that outcome data collectors were fully blinded to the study allocation may have increased the risk of courtesy or social desirability bias in participants’ responses. Second, all outcomes were self-reported and unvalidated instruments were used to assess relationship adjustment and satisfaction with care. Third, the exclusion of women advised to give birth in a referral hospital means that our findings may not be generalizable to this group. Fourth, women supplied baseline data on their male partners, whom we were unable to interview. Fifth, although very few men or couples in the control arm attended intervention sessions, their interactions in the community with participants from the intervention arm may have influenced the study’s results. This would have reduced the effect size. In fact, levels of all three primary outcomes were higher than expected in the control group, even for an urban area.^20^ However, this may have been due to undocumented secular trends. Sixth, we are unable to explain fully why certain outcomes varied across primary health centres. Anecdotal evidence suggested that there were differences between centres in how the intervention was implemented. For example, despite our efforts to ensure standardization, some staff members may have emphasized particular health messages. Finally, costing the intervention was beyond the scope of the study.

Our study was one of the first trials of a facility-based intervention to involve male partners in maternity care in sub-Saharan Africa that did not evaluate the prevention of mother-to-child HIV transmission.^17^^,^^18^ We found that even a simple educational intervention involving a maximum of three contacts can be beneficial. Our intervention could easily be replicated, or adapted for use, in similar contexts. However, because it is only possible to issue invitations if women attend health-care facilities, good intervention coverage can only be achieved where antenatal care is well attended and facility delivery is common. Elsewhere, additional community components may be necessary.^48^ Our preparatory work suggested that the involvement of male partners in routine care would be difficult in Burkina Faso because of structural and cultural constraints. However, this could be a long-term goal. 

It is important to bear in mind that policy recommendations for health workers to involve male partners may be interpreted by some as an obligation.^10^^,^^49^ As a result, male involvement may have an ambiguous effect on women’s autonomy.^12^ During our study, training and supervision ensured that health-care providers did not pressurize women to involve their partners if they did not want to. National programmes must include similar safeguards and avoid performance-based incentives. In addition, certain parts of this intervention, notably the group session for men, attempted to stimulate critical reflection on patriarchal norms. Components designed to promote equitable gender relations should be embedded in all future programmes involving men.^15^^,^^50^ In conclusion, involving men as supportive partners in maternity care can improve adherence to recommended healthy practices, with implications for family health and well-being.
